# Deciphering transcription factors and their corresponding regulatory elements during inhibitory interneuron differentiation using deep neural networks

**DOI:** 10.3389/fcell.2023.1034604

**Published:** 2023-02-20

**Authors:** Rawan Alatawneh, Yahel Salomon, Reut Eshel, Yaron Orenstein, Ramon Y. Birnbaum

**Affiliations:** ^1^ Department of Life Sciences, Faculty of Natural Sciences, Ben-Gurion University of the Negev, Beer-Sheva, Israel; ^2^ The Zlotowski Center for Neuroscience, Ben-Gurion University of the Negev, Beer-Sheva, Israel; ^3^ School of Electrical and Computer Engineering, Ben-Gurion University of the Negev, Beer-Sheva, Israel; ^4^ Department of Computer Science, Bar-Ilan University, Ramat Gan, Israel; ^5^ The Mina and Everard Goodman Faculty of Life Sciences, Bar-Ilan University, Ramat Gan, Israel

**Keywords:** non-active enhancers, repressed enhancers, deep-learning, convolution neuronal networks, predicted TF motifs, inhibitory interneuron progenitors

## Abstract

During neurogenesis, the generation and differentiation of neuronal progenitors into inhibitory gamma-aminobutyric acid-containing interneurons is dependent on the combinatorial activity of transcription factors (TFs) and their corresponding regulatory elements (REs). However, the roles of neuronal TFs and their target REs in inhibitory interneuron progenitors are not fully elucidated. Here, we developed a deep-learning-based framework to identify enriched TF motifs in gene REs (eMotif-RE), such as poised/repressed enhancers and putative silencers. Using epigenetic datasets (e.g., ATAC-seq and H3K27ac/me3 ChIP-seq) from cultured interneuron-like progenitors, we distinguished between active enhancer sequences (open chromatin with H3K27ac) and non-active enhancer sequences (open chromatin without H3K27ac). Using our eMotif-RE framework, we discovered enriched motifs of TFs such as ASCL1, SOX4, and SOX11 in the active enhancer set suggesting a cooperativity function for ASCL1 and SOX4/11 in active enhancers of neuronal progenitors. In addition, we found enriched ZEB1 and CTCF motifs in the non-active set. Using an *in vivo* enhancer assay, we showed that most of the tested putative REs from the non-active enhancer set have no enhancer activity. Two of the eight REs (25%) showed function as poised enhancers in the neuronal system. Moreover, mutated REs for ZEB1 and CTCF motifs increased their *in vivo* activity as enhancers indicating a repressive effect of ZEB1 and CTCF on these REs that likely function as repressed enhancers or silencers. Overall, our work integrates a novel framework based on deep learning together with a functional assay that elucidated novel functions of TFs and their corresponding REs. Our approach can be applied to better understand gene regulation not only in inhibitory interneuron differentiation but in other tissue and cell types.

## Introduction

The human cortex plays critical roles in cognition, motor function, and emotion (Hensch, 2005). The cerebral cortex comprises complex neuronal networks produced by two major cell types: the excitatory glutamatergic projection neurons (pyramidal cells) and gamma-aminobutyric acid-containing (GABAergic) interneurons (Whittington and Traub, 2003). Pyramidal neurons are the primary neural cells that specialize in transmitting information between different cortical regions and different brain regions. Although interneurons represent a minority (∼20%) of the entire neocortical neuronal population, they play vital inhibition roles in neuronal circuits and the cerebral cortex (Whittington and Traub, 2003). In addition, the inhibitory function of the interneurons shapes the responses of pyramidal cells and prevents runaway excitation that is required for normal brain function (Hensch, 2005). In the cortex, these interneurons are derived from neural precursors generated in the ventral forebrain (telencephalon) and undergo major tangential migration to their dorsal target tissues. The ventral telencephalon is divided into three neurogenic domains, the lateral- medial- and caudal-ganglionic eminences (LGE, MGE, and CGE respectively). The medial ganglionic eminence (MGE) is a progenitor domain within the ventral telencephalon that, together with the lateral ganglionic eminence (LGE), gives rise to the basal ganglia (striatum and globus pallidus). Via tangential migration, these structures are also the source of most interneurons in the neocortex, hippocampus, and olfactory bulb.

During neurogenesis, the generation and differentiation of neurons into GABAergic or glutamatergic subtypes is partially dependent on the combinatorial activity of transcription factors (TFs) and their corresponding regulatory elements (REs). Pro-neural TFs, such as ASCL1 and NEUROG2, were found to be necessary and sufficient to initiate neurogenesis (Bertrand et al., 2002; Aydin et al., 2019). They contribute to the specification of neuronal subtype identity (Guillemot and Hassan, 2017). The molecular mechanisms by which different TFs control gene expression and coordinate neurogenesis and inhibitory interneuron differentiation have begun to be elucidated (Guillemot and Hassan, 2017; Aydin et al., 2019). However, the remaining gaps in our knowledge make it difficult to develop diagnostic and therapeutic tools for research and clinical applications.

Recent large-scale human genetic studies have demonstrated that nucleotide variants in gene REs contribute to a wide spectrum of neurodevelopmental disorders (Lowe and Reddy, 2015; Meuleman et al., 2020). Mutations in the non-coding regions of the genome that function as gene REs can be the main cause of neurological disorders, such as epilepsy and autism (Levitt et al., 2004; Brooks-Kayal et al., 2012). Studies have produced direct evidence of a critical requirement for the correct function of enhancers in brain development (Pattabiraman et al., 2014; Nord et al., 2015). Indeed, genomic studies over the past 20 years significantly advanced the characterization of active enhancers, but their mechanism of action and their ability to drive gene expression are not fully understood.

Enhancers can be found in different epigenetic states, which are associated with their activity. Active enhancers are open chromatin regions enriched in histone modifications, such as H3K27ac and H3K4me1, and they are bound by TFs and co-activators (e.g., p300 histone acetyltransferase and the Mediator complex) (Bozek and Gompel, 2020). They are actively transcribed by RNA polymerase II into enhancer RNA (eRNA) (Carullo et al., 2020). However, additional REs, such as poised/primed/repressed enhancers, silencers, and insulators also play a role in transcriptional regulation and gene expression. As the activity state of REs is dynamic and can change rapidly during differentiation, a DNA sequence which functions as an active enhancer in a cell-specific stage can switch to a repressed enhancer or silencer state to execute the desired expression program (Huang and Ovcharenko, 2022).

Current models envision that lineage-specific TFs direct the activity state of REs (Heinz et al., 2015). Mechanistically, numerous lineage-specifying TFs were found to be pioneer factors that can bind their consensus motifs on DNA wrapped around nucleosomes, suggesting that these factors are critical for initiating chromatin opening in the locus (Fernandez Garcia et al., 2019; Bozek and Gompel, 2020). Lineage TFs require cooperation with signal-dependent TFs that bind in response to the cellular environment. In this way, the sites selectively bound by signal-dependent TFs reflect the primed, accessible chromatin landscape that is specific to each cell type (Field and Adelman, 2020). Poised enhancers are marked by markers of active enhancers, H3K4me1 and P300, but also by the repressive histone mark H3K27me3, which is associated with Polycomb Repressive Complex 2 (PRC2) silencing (Crispatzu et al., 2021). Silencers and repressed enhancers that can reduce the activity of a linked promoter are enriched in H3K27me3, which is associated with the PRC2 repressive complex (Doni Jayavelu et al., 2020; Ngan et al., 2020). Like enhancers, silencers and repressed enhancers can act in a position- and orientation-independent fashion and provide binding sites that recruit regulatory factors, in this case, transcriptional repressors (Doni Jayavelu et al., 2020; Ngan et al., 2020). This suggests that repressors are actively involved in silencing by modifying the chromatin state or occluding activating factors. How cell-specific TFs and their corresponding REs, not necessarily active enhancers, control the differentiation of neuronal progenitors toward inhibitory interneurons is still an open fundamental question.

At the DNA sequence level, a TF binds a motif and by that activates the associated RE (Field and Adelman, 2020). Many computational tools were designed to solve the motif discovery problem, i.e., finding the critical TF motif in a set of REs (Koo and Ploenzke, 2020; Thibodeau et al., 2021). In general, a dataset of regulatory genomic sequences is provided as input, and the computational tool finds short (around 10 nt) statistically over-represented motifs in the dataset. More than 100 tools aim to solve this classic bioinformatics problem (Hashim et al., 2019). This large number reflects the difficulty of the motif discovery problem and the fact that there is, still, no optimal solution.

Deep learning is a new machine-learning approach that has been revolutionizing the field of machine learning. Even in molecular biology, deep learning has been applied successfully to numerous bioinformatics problems, having outperformed many state-of-the-art methods (Min et al., 2017). By applying machine-learning approaches and bioinformatic methods together with biological functional assays, we can learn features and extract motifs affecting the transcription process. As epigenetic marks are associated with REs activity, we can now implement a deep neural network to identify gene regulatory networks of human inhibitory interneurons and thus open a venue for understanding the pathogenesis of neurodevelopmental disorders, such as epilepsy and autism.

In this work, we aimed to understand the function of gene regulation during GABAergic inhibitory-like interneuron differentiation procedure. By analyzing epigenetic datasets (ATAC-seq and H3K27ac\me3 ChIP-seq) of cultured H9 human embryonic stem cells (H9-ESC) (Day 0), MGE-like progenitors (Day 26), and mature GABAergic-like interneurons (Day 39), we were able to distinguish between active and non-active enhancers in each cell differentiation stage. Using our newly developed deep-leading-based framework, we analyzed these datasets and identified motifs of TFs that play a role not only in active enhancers, but also in poised/repressed enhancers and putative silencers that were not elucidated before.

## Results

### Identifying putative regulatory elements during inhibitory GABAergic-like interneuron differentiation

**FIGURE 1:**
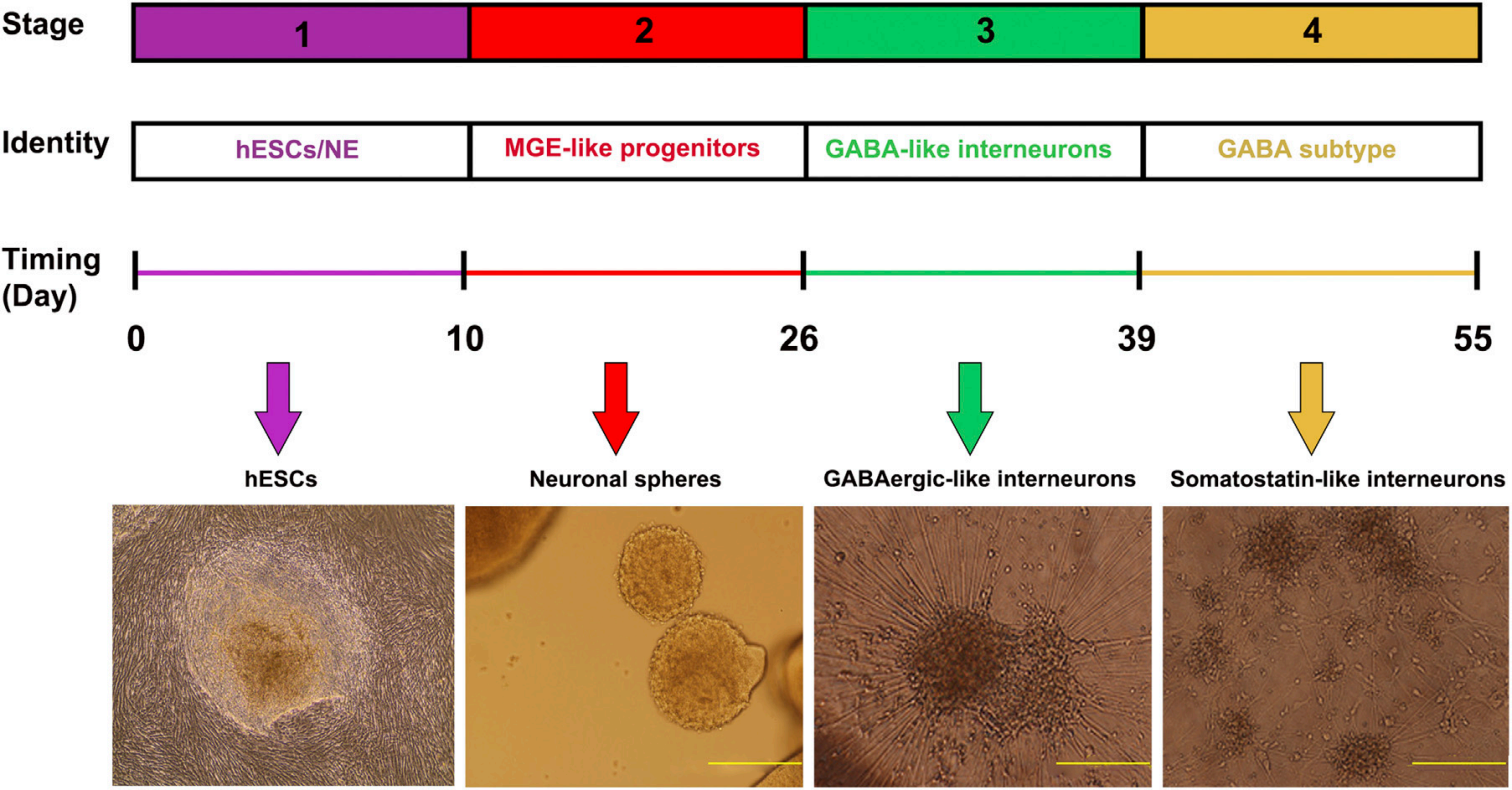
Timeline of inhibitory GABA interneuron generation. Cells are differentiated under a chemically defined system. Differentiation of H9-hESC involves 55 days of culture and four stages, including induction of neuroepithelial cells, patterning of MGE progenitors, differentiation to GABAergic-like interneurons, and eventually enriched somatostatin marked interneurons. The morphology of the cells is demonstrated in the images (Yellow bars- 500 µm).

To achieve an enriched population of inhibitory GABAergic interneurons, we cultured hESC that were differentiated into GABAergic-like interneurons based on Liu et al., 2013 (Liu et al., 2013). In brief, the GABAergic-like interneuron differentiation procedure follows four major developmental stages for 55 days culture course (Figure 1). First, the H9-hESC line is induced into primitive neuroepithelia or neural stem cells over the first 10 days. Second, the primitive neuroepithelia is patterned into ventral forebrain progenitors with the MGE feature. Third, the MGE-like progenitors are differentiated into GABAergic-like interneurons. Finally, the GABAergic-like interneurons are eventually differentiated into somatostatin (SST) subtype GABAergic-like interneurons that can be distinguished based on their neurotransmitter expression and other molecular markers, such as somatostatin (SST) and parvalbumin (PVALB). Using immunofluorescence staining, we verified the differentiation process during GABAergic-like interneurons differentiation. On day 26, the differentiated MGE-like progenitors expressed with two MGE markers of FOXG1 and NKX2-1 that were co-localized with DAPI (Supplementary Figures S1A–D). On day 39, the matured GABAergic-like interneurons expressed GAD1 and NKX2-1 (Supplementary Figures S1E–H), and further differentiation of these interneurons has characterized them as somatostatin-enriched interneurons that specifically expressed SST and SLC32A1 (Supplementary Figures S1I–L).

To identify and characterize the functional REs during neuronal differentiation, we used ATAC-seq and H3K27ac ChIP-seq data that were carried out on H9-ESC (Day 0), MGE-like progenitors (Day 26) and matured inhibitory GABAergic-like interneurons (Day 39) (Eshel et al., submitted). The H3K27ac ChIP-seq of MGE-like progenitors identified 35,000 enhancer candidates, when many of them likely regulate the expression of key TFs and epilepsy-associated genes. The peak annotation of H3K27ac from day 26 revealed that most of the peaks are promotors (45%), some of the peaks are intergenic (24%), intronic (20%), and the lower number of peaks are in protein-coding sequences (11%). Therefore, more than 50% of the peaks could be enhancer candidates that are active in MGE progenitors. As opposed to enhancer sequences, we also used epigenetic marks that are associated with repressed regions, such as H3K27me3, to identify REs other than active enhancers, such as poised enhancers and silencers. Thus, we identified novel putative REs that could control the expression of neuronal genes during differentiation.

### Determining putative active and non-active enhancers in MGE-like progenitors

**FIGURE 2:**
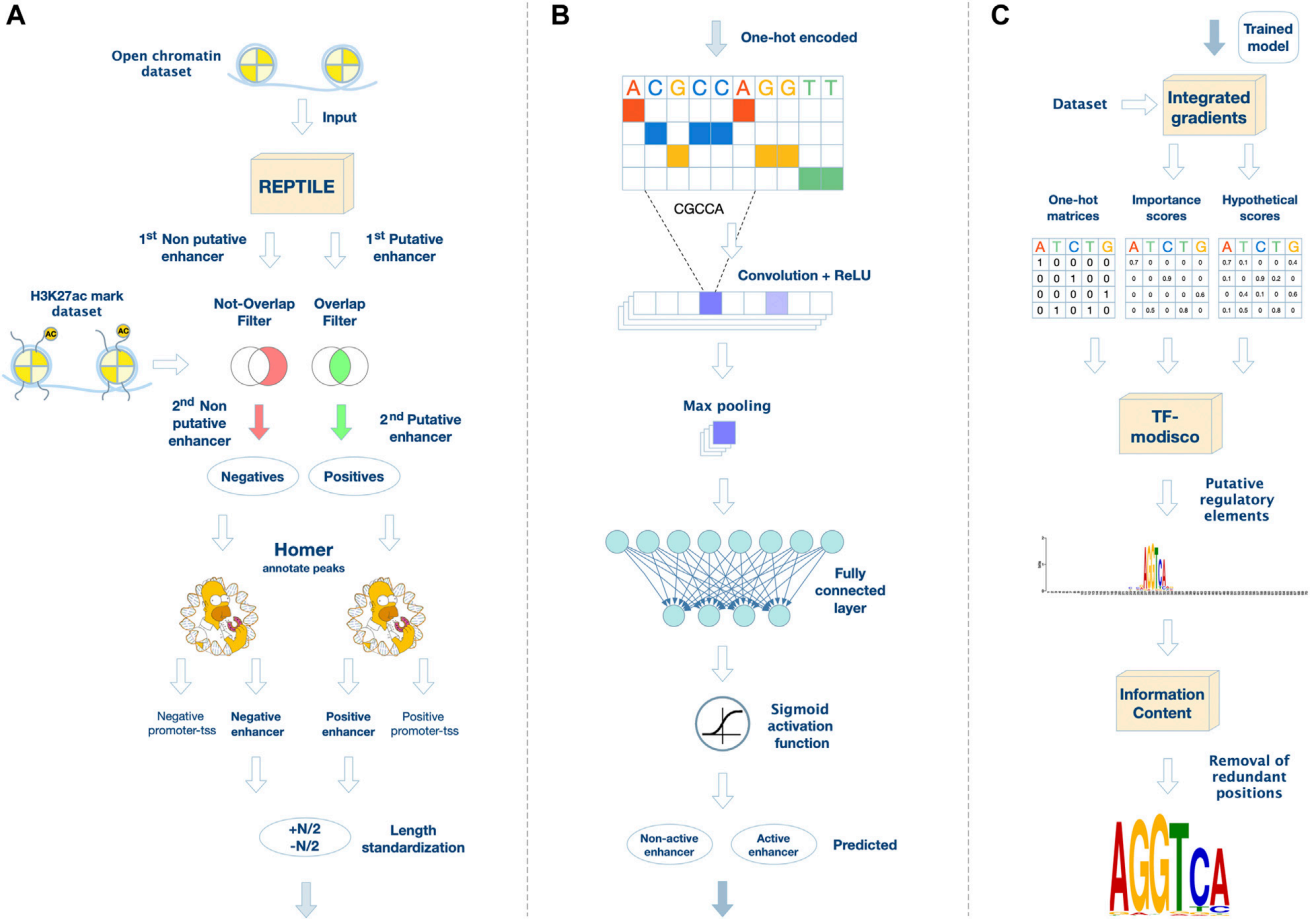
A novel framework for analyzing regulatory elements and their corresponding de novo transcription factor motifs. **(A)** Data preprocessing and filtering. We extracted putative regulatory elements from open-chromatin regions using REPTILE. Then, we generated the positive set of active enhancer regions by filtering out regions that do not overlap a H3K27ac peak in our dataset. In parallel, we generated the negative set by excluding open-chromatin regions that overlap a H3K27ac peak in our datasets. Moreover, sequences annotated by HOMER as promoter-TSS were excluded from both positive and negative sets. **(B)** CNN for active enhancer classification. The positive and negative DNA sequences are one-hot encoded as a pre-processing step to the network. In the convolutional layer, including a non-linear activation function, the filters are applied on the input matrix. Then, a max-pooling layer scans the output vector (the purple rectangle denotes the maximum value in each vector). Last, a fully connected layer models interactions, followed by an output neuron, which applies the sigmoid activation function for binary classification. **(C)** Motif extraction. We used the Integrated Gradients approach to highlight important nucleotides in each sequence. Then, we applied TF-MoDISco to aggregate these results to a list of putative motifs in PWM format. Redundant positions of the motifs were removed using an information-content criterion.

By analyzing the epigenetic dataset (H3K27ac ChIP-seq, and ATAC-seq) and the expression data (bulk RNA-seq), we aimed to elucidate the activity of gene REs during neuronal differentiation. Initially, we divided the putative REs into active enhancers (i.e., ATAC with H3K27ac peaks) and non-active enhancers (i.e., ATAC peaks without H3K27ac) for each time point. Using REPTILE (He et al., 2017), we trained our datasets to identify putative active enhancers in open chromatin regions based on the H3K27ac mark (Figure 2A). The output of REPTILE is a set of predicted active enhancers among the input open chromatin regions. Next, we extracted the putative active enhancers that overlap an H3K27ac ChIP-seq peak, and putative non-active (poised/repressed) enhancers that do not overlap H3K27ac ChIP-seq peak in our datasets (Figure 2A). Finally, 18,686 genomic regions were determined as putative active enhancers and 4,614 genomic regions as non-active enhancers on Day 26 (Supplementary Tables S1, S2).

Next, we aimed to determine whether these two sets of REs can be distinguished by specific characteristics. Enhancers exhibited a significantly high proportion of GC content and CpG islands. In the human genome, 70%–80% of CpG cytosines are methylated (Ehrlich et al., 1982; Xiong et al., 2018). Data emerging from recent genome-wide analyses suggest that active enhancers and eRNA-producing enhancers are typically hypomethylated at CpG dinucleotides (Pulakanti et al., 2013; Schlesinger et al., 2013). We analyzed the GC content of active and non-active enhancer sets. As TF motifs may be influenced by different nucleotide content, we calculated the frequency of G/C nucleotides in each sequence and found a significant difference in GC-content between active and non-active RE datasets in the three differentiation stages: Day 0, Day 26, and Day 39 (*p*-value = 5.62E-9, 2.64E-13, and 7.45E-8, respectively; Wilcoxon rank-sign test, Supplementary Figure S2).

### A novel framework for enriched motifs in regulatory elements (eMotif-RE) discovers neuronal TF motifs in putative non-active enhancers

To identify *de-novo* motifs of TFs that play a role in the activity of neuronal gene REs, we developed the motif-enriched RE (eMotif-RE) framework based on deep neural networks (Figure 2B). We performed a complete analysis of the trained models to discover TF binding sites that are enriched in putative REs. We trained a convolutional neural network (CNN) for binary classification of active and non-active enhancers to distinguish between two sets of REs, which performed much better than a linear-regression model (Supplementary Figure S3).

Then, we used TF-MoDISco to detect motifs through the datasets and trained networks (Figure 2C). Furthermore, we performed a post-analysis of the identified motifs using TOMTOM, which compares the motifs to known TF motifs (Gupta et al., 2007). Moreover, we applied the analysis of motif enrichment (AME) to test the enrichment of the identified motifs in the active and non-active enhancer datasets (McLeay and Bailey, 2010) (Supplementary Table S3, S4).

By applying our newly developed framework on our epigenetic data, we analyzed the motif enrichment and compared our framework results with established motif-finding methods, including DREME, MEME, and BaMM (Bailey et al., 2015; Siebert and Soding, 2016; Kiesel et al., 2018). We obtained a list of motifs that are enriched in the active enhancer set and showed a high similarity to known TFs (Supplementary Table S5). Moreover, we found a subset of motifs that are enriched in the non-active enhancer set and showed a high similarity to known TFs (Supplementary Table S5). As various methods for *de novo* motif finding produce putative spurious motifs, we filtered out motifs according to guideline criteria (see disqualifying spurious motifs; Materials and Methods). Finally, we ranked the motifs by the expression levels of their corresponding TFs in Day 0 and Day 26 (Supplementary Table S6).

**FIGURE 3:**
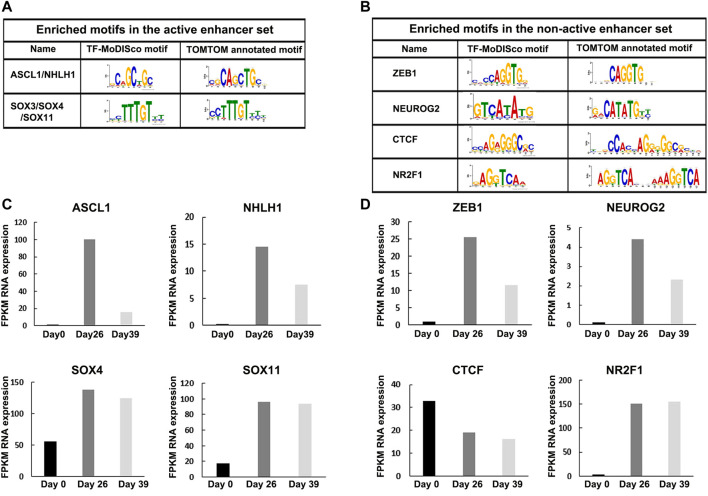
Enriched motifs in active and non-active enhancer sets with differential RNA expression levels at Day 26. **(A)** Enriched motifs in active and **(B)** non-active enhancer sets following a motif analysis using TF-MoDISco and TOMTOM. **(C)** RNA expression levels of NHLH1, ASCL1, and SOX4/11 TFs were highly expressed in the active set at Day 26. **(D)** RNA expression levels of ZEB1, NEUROG2, CTCF, and NR2F1 TFs were highly expressed in the non-active set at Day 26.

We found two enriched *de-novo* motifs in the active enhancers set: “CAGCTGC” and “CCTTTGT” (Figure 3A). The first motif (‘CAGCTGC’) is homologous to the binding site of two neuronal TFs: The Achaete-Scute Family BHLH Transcription Factor 1 (ASCL1), which plays a role in the neuronal commitment and differentiation. ASCL1 acts as a pioneer TF accessing closed chromatin to allow other factors to bind and activate neural pathways (Castro et al., 2011; Wapinski et al., 2013; Woods et al., 2022); and The Nescient helix-loop-helix 1 (NHLH1) that has a similar binding motif as ASCL1. NHLH1 is expressed in the neuroepithelium and plays a role in neuronal differentiation (Kruger and Braun, 2002). The second motif (“CCTTTGT”) is homologous to the binding site of Sox family members, including SOX4 and SOX11. Interestingly, SOX4, together with SOX11 and SOX12, forms the group C type of SRY-related TFs (Dy et al., 2008). They play key roles, often in redundancy, in multiple developmental pathways, including neurogenesis. *De novo* SOX11 heterozygous mutations have been shown to cause intellectual disability, growth deficiency, and dysmorphic features compatible with mild Coffin-Siris syndrome (Mu et al., 2012; Zawerton et al., 2019). SOX4 and SOX11 target the promoters of genes that are induced in neuronal differentiation. Moreover, ASCL1 strongly synergized with SOX4 and SOX11 in the activation of neuronal enhancers when the two TFs were overexpressed together (Minieri, 2014). We analyzed the co-occurrence of ASCL1 and SOX4/11 motifs in active and non-active enhancer sets and found that out of 13,688 ASCL1 sites and 10,334 SOX4/11 sites in active enhancers, 6,944 sites share the same active enhancer (*p*-value<10^–16^, Fisher exact test). Thus, we conclude that ASCL1 and SOX4/11 motifs are significantly co-enriched motifs in active enhancers, supporting their potential synergetic effect in activating neuronal enhancers.

We found four enriched *de-novo* motifs in the non-active enhancers set: “CCAGGTG”, “GTCATATG”, “CCAGGGGGCGA”, and “GAGGTCAA” (Figure 3B). The first motif (“CCAGGTG”) is homologous to the binding site of Zinc finger E-box-binding homeobox 1 (ZEB1), a TF that can function both as activator and repressor depending on its target gene and tissue (Zhang et al., 2019). ZEB1 is an essential factor for neocortical development, expressed in several neuronal tissues, as well as the proliferative zones in the brain and spinal cord of mouse embryos (Liu et al., 2019; Wang et al., 2019). The second motif (“GTCATATG”) is a homologous to the binding site of Neurogenin 2 (NEUROG2) which is a pro-neural factor that increases chromatin accessibility, mediates enhancer activity, and facilitates chromatin looping (Noack et al., 2022). The third motif (“CCAGGGGGCGA”) is a homologous to the binding site of CCCTC-binding factor (CTCF), a highly conserved zinc-finger protein that functions as a transcriptional activator, repressor, or insulator protein, blocking the communication between enhancers and promoters (Kim et al., 2015). The fourth motif (“GAGGTCAA”) is a homologous to the binding site of (NR2F1), coding for a transcriptional regulator belonging to the steroid/thyroid hormone receptor superfamily that is known to play key roles in several brain developmental processes, from proliferation and differentiation of neural progenitors to migration and identity acquisition of neocortical neurons (Tocco et al., 2021). In comparison, competing motif analysis methods found only spurious motifs or detected only the ZEB1 motif among a long list of spurious motifs (Supplementary Figure S4; Supplementary Table S5).

Next, we analyzed the expression level of these motif-enriched TFs in hESC (Day 0), MGE-like progenitors (Day 26), and mature GABAergic-like interneurons (Day 39). Using RNA-seq, we found that the expression levels of the TFs with enriched motifs in the active set (i.e., ASCL1, NHLH1, SOX4, and SOX11) were elevated during differentiation (Day 0 vs. Day 26) (Figure 3C), and the expression levels of the TFs with enriched motifs in the non-active set (i.e., ZEB1, NEUROG2, CTCF, and NR2F1), were also elevated during differentiation, except for CTCF that is highly expressed in the three differentiation stages (Figure 3D). Thus, the identified motif-enriched TFs from eMotif-RE correlate with their expression levels, supporting their regulatory activity.

### Putative regulatory elements function as *in vivo* active and poised enhancers

**FIGURE 4:**
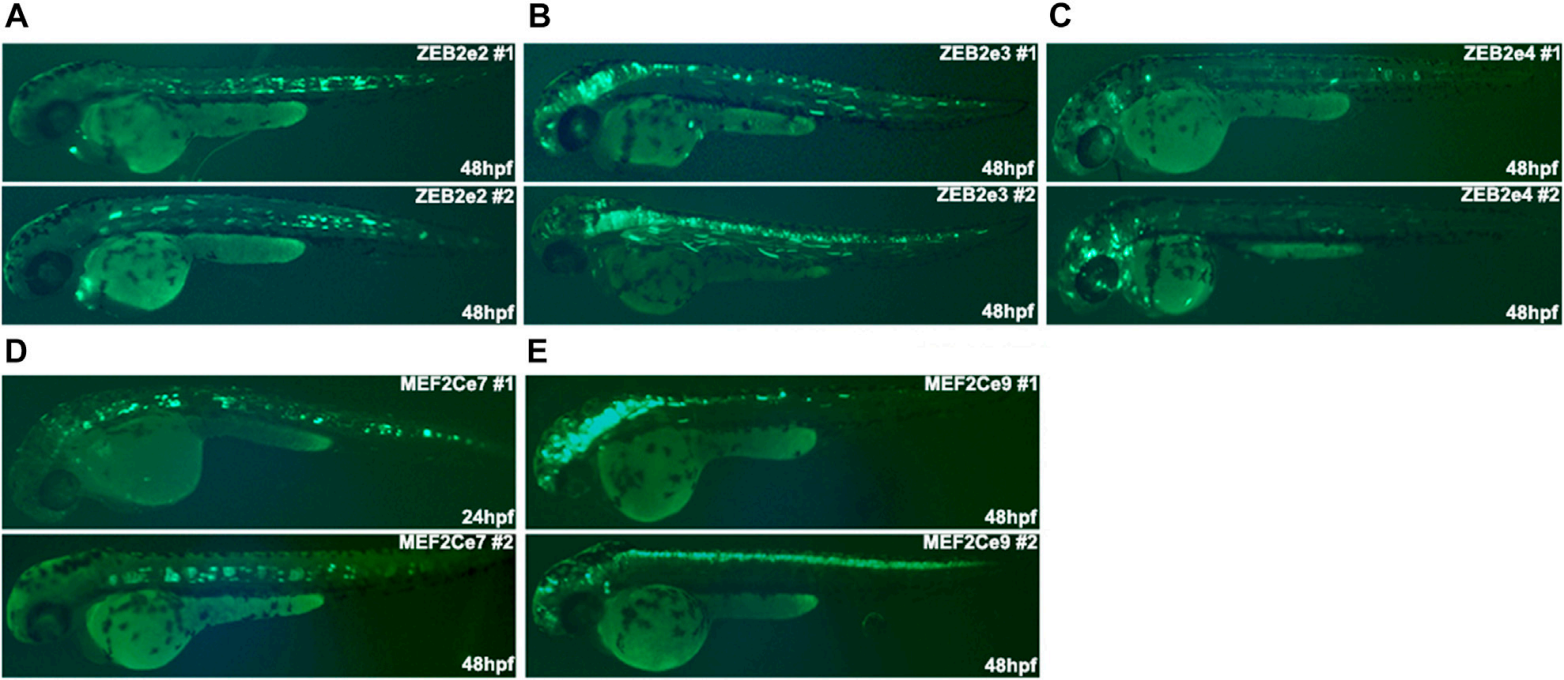
The *in vivo* activity of regulatory elements marked as active enhancers at 24/48 hpf zebrafish embryos. **(A)** ZEB2e2 drove GFP expression in the notochord. **(B)** ZEB2e3 drove GFP expression in the midbrain, hindbrain, spinal cord, and somitic muscles. **(C)** ZEB2e4 drove GFP expression in notochord and non-specific neurons. **(D)** MEF2Ce7 drove GFP expression in the notochord. **(E)** MEF2Ce9 drove GFP expression in the midbrain, hindbrain, and spinal cord. The pattern of each enhancer is represented by images of two independent transgenic zebrafish embryos.

To characterize the *in vivo* activity of the putative REs, we used an enhancer assay in zebrafish, which is a rapid and cost-effective assay to determine the spatiotemporal enhancer activity. We selected several putative REs that marked as active and non-active enhancers in cultured human MGE-like progenitors of Day 26 (Table 1). Moreover, the selected putative active enhancers are located near highly expressed gene/s at Day 26 and the selected non-active enhancers are located near genes that are differentially expressed at Day 26. We amplified these putative REs from human genomic DNA and cloned them into a zebrafish enhancer assay vector, containing an E1b minimal promoter followed by the green fluorescent protein (GFP) reporter gene. These vectors were microinjected into one-cell stage zebrafish embryos along with the Tol2 transposase to facilitate genomic integration. The transgenic embryos were monitored for GFP expression at 24-48 hours post fertilization (hpf). We have previously shown that putative REs that are marked as active enhancers drove consistent GFP expression (≥30% of GFP expressed embryos) in specific neuronal tissues (Bar Yaacov et al., 2019; D'Haene et al., 2019). Neuronal-specific enhancers of *ZEB2* and *MEF2C* drove GFP expression in the brain, notochord, and spinal cord that resemble the expression of these two genes (Figure 4; Table 1) (Bar Yaacov et al., 2019; D’Haene et al., 2019). Moreover, the zebrafish orthologous TFs with the enriched motifs in active and non-active REs (such as ASCL1, ZEB1, and CTCF) are evolutionarily conserved with an average of 60% identity (48%–76%) and 72% (62%–83%) similarity to human protein sequences (Supplementary Table S7). Thus, open chromatin regions marked by H3K27ac function as active enhancers, but the function of non-active enhancer regions are barely investigated.

**TABLE 1:** Enhancer activity of selected putative regulatory elements using zebrafish enhancer assay.

		Human hg38 assembly			ATAC-seq signal	H3K27ac ChIP-seq signal
Name	Expression patterns of the enhancer activity	Chr.	Start	End	Day 26	Day 26
*ZEB2e2*	Notochord	chr2	144430328	144431310	+	+
*ZEB2e3*	Midbrain, hindbrain, spinal cord, somitic muscles	chr2	144430502	144432268	+	+
*ZEB2e4*	Notochord, non-specific neurons	chr2	144438728	144440073	+	+
*MEF2Ce7*	Notochord	chr5	89703170	89704367	+	+
*MEF2Ce9*	Midbrain, hindbrain, spinal cord	chr5	89822526	89823501	+	+
*RE1*	Negative	chr12	54993195	54993747	+	-
*RE2*	Negative	chr12	54715951	54716496	+	-
*RE3*	Specific neurons in the forebrain, Somitic muscles	chr14	36604850	36605388	+	-
*RE4*	Negative	chr2	44466627	44467238	+	-
*RE5*	Negative	chr1	27349403	27350221	+	-
*RE6*	Negative	chr19	6753176	6753722	+	-
*RE7*	Negative	chr1	16226632	16227187	+	-
*RE8*	Forebrain, specific neurons around the eye	chr6	126143965	126144514	+	-

**FIGURE 5:**
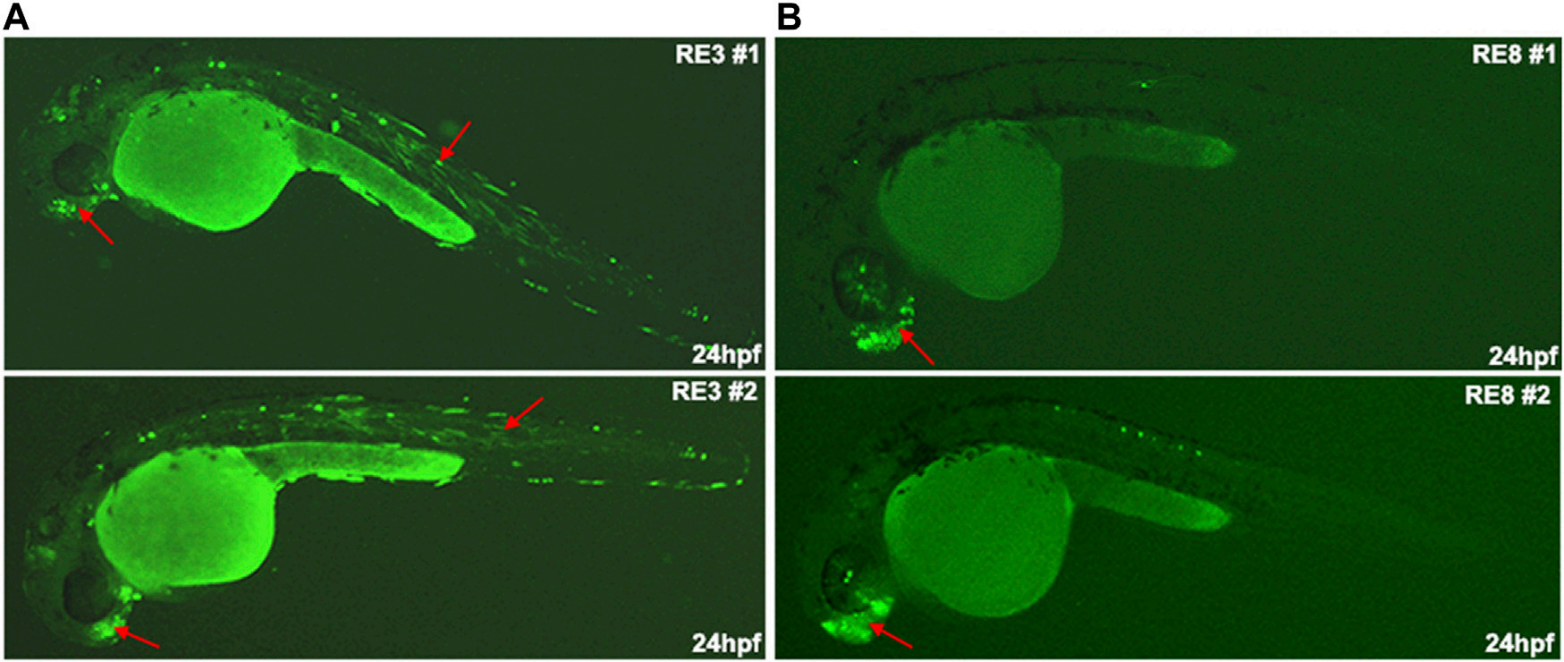
The *in vivo* enhancer activity of regulatory elements (REs) from the non-active enhancer set. **(A)** RE3 drove GFP expression in specific neurons in the forebrain and somitic muscles at 24 hpf zebrafish embryos. **(B)** RE8 drove GFP expression in the forebrain at 24 hpf zebrafish embryos (red arrows in biological replicates).

As the open chromatin regions that are not marked as active enhancers may function as poised enhancers, we tested if these regions could function as neuronal enhancers at later stages of differentiation. We selected eight putative REs located near genes that are highly expressed in Day 0 compared to Day 26 (such as *STYL1, TRIP10, ARHGEF19,* and *CENPW*) or near genes that are not expressed in Day 0 but have high expression levels in Day 26 (such as *NEUROD4, NKX2-1/8, UNCX,* and *SIX3*). Moreover, the selected REs are enriched with predicted binding sites for ZEB1, CTCF, and NEUROG2 that likely play a repressive role in regulating these elements. We found that two out of the eight tested putative REs (RE3 and RE8) showed neuronal enhancer activity, while no enhancer activity was observed for the other six putative REs (Table 1). RE3 drove GFP expression in specific neurons of the forebrain and in somitic muscles (Figure 5A), and RE8 drove GFP expression in the forebrain, specific neurons above the eye, and along the developing body of zebrafish embryos at 24 hpf (Figure 5B). Thus, these two REs likely function as poised enhancers during development, but most of the selected REs did not show enhancer activity in the zebrafish assay suggesting that they might function as repressed enhancers or silencers (Table 1; Supplementary Table S8).

### Deletions of ZEB1 and CTCF predicted binding motifs increased the activity of their targeted regulatory elements

**FIGURE 6:**
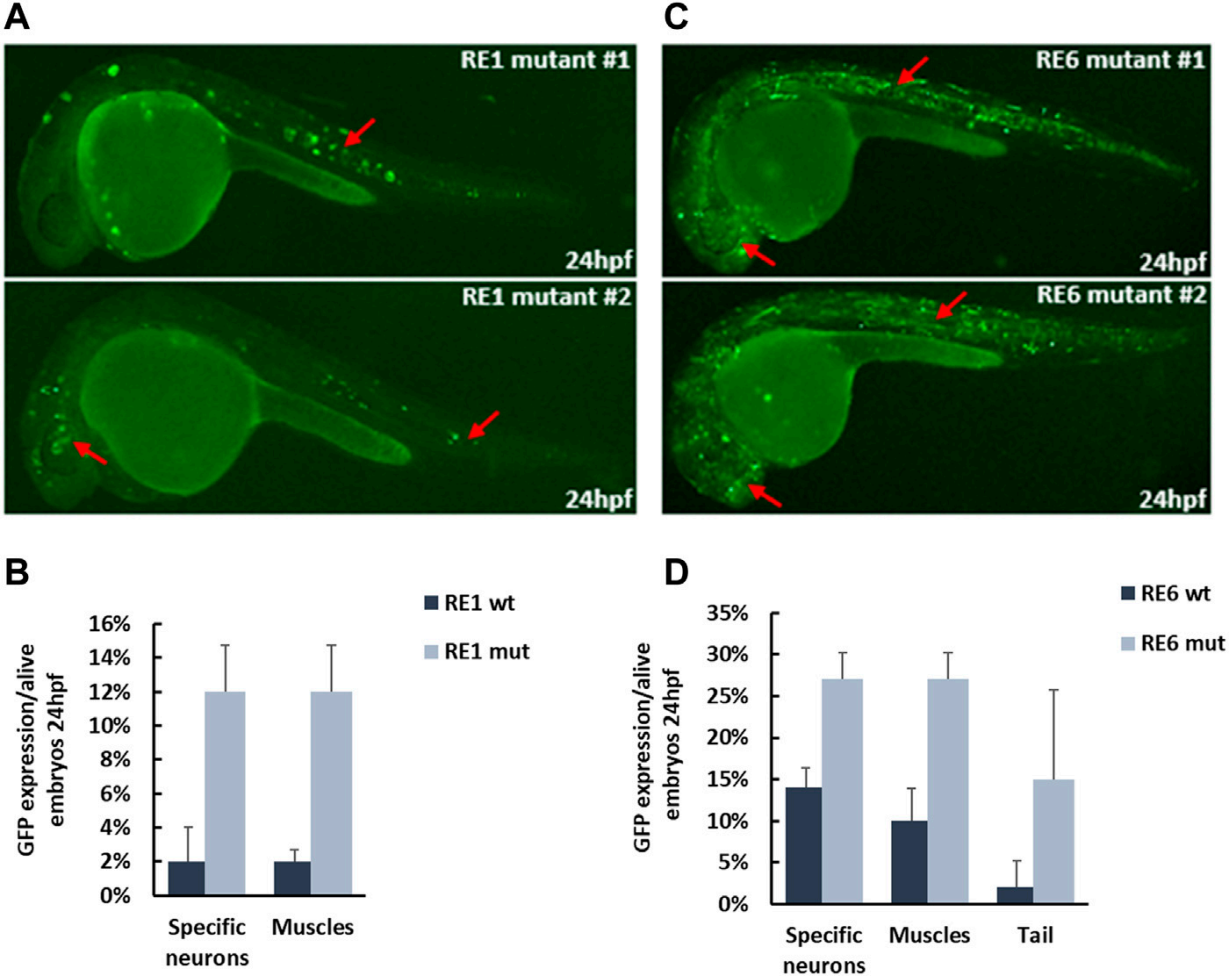
*In vivo* activity of repressed enhancers at 24 hpf zebrafish embryos. **(A)** RE1 mutant sequence drove GFP expression in specific neurons in the brain and around the eyes and notochord (red arrows). **(B)** Six-fold increase in the number of positive zebrafish embryos with the RE1 mutant sequence compared to the reference sequence. **(C)** RE6 mutant sequence drove GFP expression in forebrain and somitic muscles (red arrows). **(D)** Two-fold increase in the number of positive zebrafish embryos with the RE6 mutant sequence compared to the reference sequence.

To test whether the selected REs function as repressed enhancers or silencers, we deleted the binding sites of ZEB1 and CTCF from the REs and tested their activity as enhancers. Since ZEB1 and CTCF can function as transcription repressors, we selected RE1 and RE6, as two putative REs that are enriched for ZEB1 and CTCF binding motifs. We deleted the two predicted ZEB1 binding sites from RE1 and the CTCF binding site that overlaps with a ZEB1 binding site (Supplementary Figure S5). We also deleted the three ZEB1 binding sites and a CTCF binding site from RE6 (Supplementary Figure S5). Next, we tested the activity of RE1 and RE6 mutants using a zebrafish enhancer assay. While RE1 and RE6 did not function as active enhancers *in vivo* (<30% of live embryos), the RE1 and RE6 mutants drove GFP expression in neuronal tissues with a higher number of positive GFP embryos. RE1 mutant embryos drove GFP expression in specific neurons in the brain and the notochord with a 6-fold increase compared to the reference sequence (Figures 6A, B). RE6 mutant embryos drove GFP expression in the forebrain and somitic muscles with a 2-fold increase compared to the reference sequence (Figures 6C, D). Thus, our results show that deletions of ZEB1 and CTCF binding sites increase the RE1 and RE6 activity in this assay suggesting that these open chromatin regions might function as repressed enhancers.

## Discussion

In this study, we deciphered the activity of REs enriched for motifs of specific TFs that play a role in their spatiotemporal activity during neuronal differentiation. We developed a novel framework for *de novo* motif finding (eMotif-RE), which utilizes recent advancements in deep neural networks for motif-finding tools via interpretation of CNNs predictions (Lundberg Scott, 2017; Maslova et al., 2020; Ting Zhang et al., 2021). By focusing on finding motifs of TFs that control the activity of neuronal gene REs, we analyzed unique genomic datasets, which encompass open-chromatin regions (ATAC-seq) and active enhancer regions (H3K27ac ChIP-seq) during neuronal differentiation. Using REPTILE, we pre-processed these datasets to generate two confident sets of active and non-active enhancers. We took advantage of the capabilities of CNNs, which have shown great success in many bioinformatic challenges in recent years (Barshai et al., 2020; Zeng et al., 2020; He et al., 2021), to learn the important features in sequence data to predict whether a DNA sequence belongs to an active or non-active enhancer region. Moreover, with the trained models and sequence datasets, we used the Integrated Gradient method to highlight the important features in every sample in the dataset and aggregated the results by TF-MoDISco to extract putative regulatory motifs. In TF-MoDISco, the output motifs include redundant positions and spurious motifs. Therefore, we used information-based criteria to remove redundant positions and filter spurious motifs. Furthermore, we examined the gene expression (RNA-seq) levels of the TFs with enriched binding sites. This test allowed us to verify that the identified TFs are indeed important in neuronal cells. We performed another statistical analysis using the AME tool to verify the enrichment of the putative motifs in the sequence datasets.

We tackled a couple of limitations in our eMotif-RE framework. First, as TF-MoDISco does not provide statistical information on the identified motifs, we used MEME-suite statistical tools and defined unique guideline criteria to filter out irrelevant motifs. Second, it is possible that under other hyper-parameters values of the CNN we would have obtained different motifs. We solved this issue by using grid-search to find the optimal hyper-parameters. The disadvantage of using grid search is the high run-time, which limits the parameter space that can be searched.

The outcome of the eMotif-RE analysis revealed that ASCL1 and SOX4/SOX11 are enriched in active enhancers of inhibitory interneuron-like progenitors. Both ASCL1 and SOX4/SOX11 are known to function as TFs in neuronal differentiation, but our analysis suggests that they function together by regulating the activity of specific enhancers in neuronal progenitors as we found significant co-enrichment of the two motifs in active enhancers. ASCL1 functions as a pioneer TF accessing closed chromatin to allow other factors to bind and activate neural pathways (Castro et al., 2011; Aslanpour et al., 2020; Woods et al., 2022) and SOX4/SOX11 induce the activity of neuronal differentiation by regulating promoters. Moreover, over-expression of ASCL1 and SOX4/SOX11 showed a synergetic effect on the activation of neuronal enhancers (Minieri, 2014) supporting that these TFs are likely working together.

Our results showed that the ASCL1 motif is enriched in active enhancers, while the NEUROG2 motif is enriched in the poised/primed/repressed REs. Therefore, these two factors, ASCL1 and NEUROG2, might have an opposite regulatory effect on the REs of inhibitory interneuron progenitors. Indeed, direct neuronal programming of embryonic stem cells showed that these two main vertebrate pro-neural factors, ASCL1 and NEUROG2, bind to largely different sets of genomic sites to induce different neuronal fates (Aydin et al., 2019). While ASCL1 binds and activates enhancers that are required for the differentiation of GABAergic inhibitory interneurons, NEUROG2 is required for differentiation toward glutamatergic neurons. Our data suggest that during differentiation of GABAergic inhibitory interneurons, ASCL1 binds and activates the desired enhancers and NEUROG2 might play a role in regulating primed/repressed REs, which constrain terminal cell fates and enforces the differentiation toward GABAergic interneurons and not glutamatergic neurons.

ZEB1 and CTCF motifs were enriched in the non-active enhancer set. Both ZEB1 and CTCF can function as repressors and inhibit enhancer activity required for regulating the transcriptional program. ZEB1 is an essential factor for neocortical development, expressed in several neuronal tissues, as well as the proliferative zones in the brain and spinal cord of mouse embryos (Liu et al., 2019; Wang et al., 2019). CTCF is mainly known as a chromatin remodeler and insulator to define loops and TAD boundaries, but it is also known to function as a TF to regulate gene expression (Kim et al., 2015; Nora et al., 2017). Indeed, we showed that deletions of the ZEB1 and CTCF predicted binding sites in RE1 and RE6 induced the number of GFP-positive zebrafish embryos (Figure 6). The elevated activity of the mutated RE1 and RE6 supports the repressive effect of ZEB1/CTCF on REs that require to maintain their repression.

Finally, we classified the REs into active and non-active enhancer sets. While the active enhancer set is well defined, the non-active enhancer set that is characterized by open chromatin can be divided into additional classes according to their activity. We showed that they can function as poised enhancers that are not active at progenitor cells (Day 26) but are likely to become active at later stages of neuronal differentiation, such as RE3 and RE8. These two REs are marked as open chromatin but showed function as neuronal enhancers at only later stages of development (Figure 5). Moreover, REs can also function as repressed enhancers/silencers as deletions of transcription repressor binding sites, such as in RE1 and RE6, elevated their activity as enhancers (Figure 6). The other tested putative REs that did not show enhancer activity might function as different REs, such as silencers or insulator elements.

In this study, we analyzed human sequences for their *in vivo* enhancer activity using a zebrafish enhancer assay. Given that human sequences are tested for enhancer activity in the zebrafish model, one potential pitfall is that they may not be functional in zebrafish. This assay is not a high-throughput functional analysis of REs and the tested human sequences portray regulatory activity even if they do not have homologous sequences in zebrafish. Human sequences that did not show activity in this assay may have enhancer activity in the spatiotemporal endogenous context when the required TFs and additional associated proteins are expressed.

To conclude, our development of eMotif-RE framework allowed us to identify *de-novo* motifs in various REs and elucidate novel roles for these TFs in regulating neuronal transcription patterns. Our eMotif-RE framework emphases the interplay between TFs and various classes of REs to execute the spatiotemporal expression programs required for neuronal differentiation and normal brain development. Disruption of these various classes of active/poised/repressed/silenced REs may lead to mis-expression with the outcome of neurodevelopmental disorders. Further functional studies are needed to explore the molecular mechanism and function of REs such as poised/repressed enhancers or silencers, specifically during neuronal differentiation.

## Materials and methods

### Data pre-processing and filtering

#### Dataset source

We used datasets encompassing open chromatin (ATAC-seq) and active enhancers (H3K27ac ChIP-seq) experimental datasets of H9-hESC (Day 0), MGE-like progenitors (Day 26), and inhibitory-like interneurons (Day 39). We used Model-based Analysis of ChIP-Seq (MACS) (Feng et al., 2012) for peak calling to identify open chromatin and H3K27ac-enriched genomic regions based on raw sequencing files (GEO; accession number GSE218668). Then, we identified active and non-active regions using REPTILE which locates enhancers based on genome-wide DNA methylation and histone modification profiling (He et al., 2017). As methylation data was not available in our study, we only used the H3K27ac epigenetic mark, which is associated with active enhancers. We trained REPTILE on ChIP-seq experiments conducted in mouse embryonic stem cells, which were provided as example files with the REPTILE software package. This training data included a H3K27ac ChIP-seq dataset in bigwig format, and a ground truth file with annotations of active and non-active enhancers. We trained REPTILE to identify active enhancers in open chromatin regions based on the H3K27ac mark alone (Figure 2A). The output of REPTILE is a set of predicted active enhancers among the input open chromatin regions. From the regions defined by REPTILE, we further extracted the putative active enhancers that overlap an H3K27ac ChIP-seq peak, and putative non-active (poised/repressed) enhancers that do not overlap any H3K27ac ChIP-seq peak (Figure 2A). To extract the sequences corresponding to the genomic coordinates, we used BEDTools (Quinlan and Hall, 2010), an efficient tool to analyze and process large genomic datasets. Since deep neural networks require fixed-size samples, we set all sequence lengths to be the length of the shortest sample size in the set. For length N, we selected N/2 nucleotides upstream and downstream of the center of each peak (Figure 2A). We set the sample size of the dataset to the shortest sample size, which was 500 nt for Day 26 and Day 39 and 101 nt for Day 0.

#### Enhancer annotation by Homer

We used Homer (Heinz et al., 2010), a toolkit for motif discovery and next-generation sequencing analysis, for enhancer annotation. Homer includes a script for genomic annotation to any genomic coordinate. We used annotatePeaks.pl to remove sequences that regulate the transcription process and are adjacent (−1,000, 100) to the transcription start site (TSS). These control sequences are annotated as “promoters-TSS” in the output of the script.

#### Deep neural network architecture

We developed a CNN for the binary classification of active or non-active enhancers (Figure 2B). The active enhancer set is the positive set, and the non-active enhancer set is the negative set. The network architecture was inspired by common CNNs in genomics (Zeng et al., 2016). The network receives a single type of data as input, a DNA sequence of length *L*. Each nucleotide is encoded as a one-hot vector of dimension *d* = 4. The first layer of the network is a 1D-convolutional layer. A rectified linear unit, *f(x)* = max (0, *x*), is applied as a non-linear activation function on the convolution output. The max-pooling layer scans the output vector of each filter and outputs the maximum value in it. A mid-level flattening is required to get an output vector composed of all maximum filter outputs. A fully connected layer computes a weighted sum of the input from the previous layer. Network training and testing, including evaluation of prediction performance and hyper-parameters search, are described in Supplementary Information.

#### Motif extraction by TF-MoDISco

TF-MoDISco (Avanti Shrikumar, 2018) was developed to identify short motifs (around 10 nt) given a sequence dataset, importance scores, and hypothetical importance scores associated with each sequence in the dataset as calculated over a trained neural network. To obtain the importance scores and hypothetical importance scores for each sequence, we used the Integrated Gradients method (Sundararajan et al., 2017). Integrated Gradients receives as input a sequence and a trained model, and outputs importance scores for each nucleotide in the sequence based on the trained network. The hypothetical importance scores inform what the importance scores would be for nucleotides, other than the ones in the given sequence. TF-MoDISco combines the contribution of multiple pattern detectors and extracts important sequence features. The output of TF-MoDISco is a list of motifs in the form of position weight matrices (PWMs) (Figure 2C). Technically, the output comprises three files for each motif: i) a motif pattern PWM, ii) a motif importance score matrix, and iii) a motif hypothetical importance score matrix. A positive motif importance score means that the motif is enriched in the positive set, and a negative motif importance score means the motif is enriched in the negative set. TF-MoDISco outputs 70 nt-long motifs, but most motif lengths, that represent a TF binding site, are approximately 10 nt-long. Hence, the output of each motif contains around 60 redundant positions, which are typically characterized by high entropy (Figure 2C). Unfortunately, there is no known optimal entropy threshold for removing redundant positions in a PWM (Pan and Phan, 2008). Therefore, we used the information-content (IC) criterion to remove redundant positions (Eq. 1). The first position with a score of over 0.3 is the start of the motif, and the first position from the other side is the end of the motif. Denote *P_j_* the *j*-th column of PWM *P*, and by *P*_i,__*j*_ the probability having nucleotide *i* at position *j*. The information content of *P_j_* is defined as:

$$IC\;\left(P_{j}\right)=2+\underset{i=\left\{A,C,G,T\right\}}{\sum }P_{i,j}\log_{2}P_{i,j}$$ (1)

### Motif post-analysis

#### Applying MEME-suite for motif enrichment and similarity analysis

We used AME (McLeay and Bailey, 2010) to test the enrichment of a given motif in an active enhancer compared to a non-active enhancer set. We used TOMTOM (Gupta et al., 2007) to calculate the similarity of a given motif to a database of target motifs. Technical details of how MEME-suite methods were used are in Supplementary Information.

#### Calculating PWM stringency

Some of the irrelevant motifs detected by the motif-finding tools can be easily excluded by calculating their stringency. Each position in a PWM represents the preference for each one of the nucleotides A, C, G, and T. It is unlikely that a PWM of TF will be represented as a stringent motif (Weirauch et al., 2013). We define a stringent position by IC > 1.9 bits.

#### Disqualifying spurious motifs

The various methods for *de novo* motif finding produce a list of putative motifs. Within those lists, there are some spurious motifs, which can be identified by different criteria. We defined multiple criteria to filter out spurious motifs. We kept the motifs that passed the following criteria.

1) Bias <80% (GC-rich test, GC/AT percentage of the most preferred nucleotides in each position does not exceed 80%)
2) Length >4 nt
3) Stringent <75% (the number of stringent positions is less than 75%)
4) Correspond (the motif corresponded to at least one known TF motif as detected by TOMTOM)
5) Overlap >70% (the motif must align with an overlap of at least 70% to at least one known TF motif as detected by TOMTOM)
6) q-value <0.05 (TOTOM motif similarity of significance)
7) Differential expression of TF in neuronal progenitors (2-fold change)

### Transposase-accessible chromatin with high throughput sequencing (ATAC-seq)

ATAC-seq is a technique used in molecular biology to assess genome-wide chromatin accessibility (Buenrostro et al., 2013). ATAC-seq is a faster and more sensitive analysis of the epigenome than DNase-seq or MNase-seq. ATAC-seq was performed on MGE-like progenitors (Day 26) and GABAergic-like interneurons (Day 39) in duplicates. The cells were counted, and 50,000 cells were taken for each experiment. Cells were washed with cold PBS and were lysate. Immediately after lysis the nuclei were taken to transposition reaction using Nextera Tn5 Transposase, (Illumina Cat.FC-121-1030) and incubated at 37°C for 30 min, with gentle mixing. After the transposition reaction, the pellet was purified using a Qiagen MinElute PCR Purification kit (Qiagen cat. 28004, Germany). The purified transposed DNA fragments were amplified using NEBNext High-Fidelity 2X PCR Master Mix (New England Labs Cat.M0541, United States) with 25uM PCR Primer 1 (AAT​GAT​ACG​GCG​ACC​ACC​GAG​ATC​TAC​ACT​CGT​CGG​CAG​CGT​CAG​ATG​TG), Barcoded PCR Primer 2 (CAA​GCA​GAA​GAC​GGC​ATA​CGA​GAT​TCG​CCT​TAG​TCT​CGT​GGG​CTC​GGA​GAT​GT, CAA​GCA​GAA​GAC​GGC​ATA​CGA​GAT​CTA​GTA​CGG​TCT​CGT​GGG​CTC​GGA​GAT​GT). The PCR program was as published in the protocol: 1 cycle of 5 min 72°C, 30 s 98°C followed by 5 cycles of 10 s 98°C, 30 s 63°C, 1 min 72°C. To reduce bias to size and GC content the PCR must be stopped before saturation. Therefore, a qPCR side reaction was done to determine the number of PCR cycles to add. A 5 μL of previously PCR-amplified DNA using NEBNext High-Fidelity 2× PCR Master Mix and 100× SYBR Green I the fragment were amplified in qPCR using the protocol: 1 cycle of 30 s 98°C and 20 cycles of 10 s 98°C, 30 s 63°C, 1 min 72°C. The cycle number 6 showed one-third of the maximum fluorescent intensity is the cycle to add to the PCR and therefore it was chosen for the following reaction. The remaining PCR reaction (45 μL) was amplified in a second PCR using the program: 1 cycle of 30 s 98°C and 6 cycles of 10 s 98°C, 30 s 63°C, 1 min 72°C. The libraries were purified using Qiagen MinElute PCR Purification Kit. The concentration of the purified libraries was calculated using Qubit and Bioanalyzer. The amplified libraries were sequenced by Next-seq for pair-end reads with a coverage of 40 M reads per sample.

### Chromatin immunoprecipitation followed by sequencing (ChIP-seq)

ChIP-seq is a method used to analyze protein interactions with DNA. ChIP-seq combines with massively parallel DNA sequencing to identify the binding sites of DNA-associated proteins. 10^6^ differentiated cells from Day 26, Day 39, and Day 55 stages were cross-linked using 1% formaldehyde. The lysate with sodium dodecyl sulfate-based reagents and chromatin was sonicated for 18 cycles (60 s On, 60 s Off) using Bioruptor. The sonicated samples were immunoprecipitated using magnetic beads 25 μL protein A (Invitrogen cat.10002D) and 25 μL protein G (Invitrogen cat.10004D). The samples were reverse crosslinked using Proteinase K overnight at 650°C. The sonicated fragments were 300-500bp in size. The DNA fragments were purified using the phenol-chloroform protocol. ChIP was performed using antibodies against H3K27ac (Abcam Ab4729) and H3K27me3 (Abcam Ab4729). Prepared libraries from ChIP and input DNA were sequenced using the HiSeq instrument (Illumina, United States). The ChIP libraries were analyzed and mapped to hg19 using BWA (Li and Durbin, 2009) and peaks were called using MACS (Zhang et al., 2008).

### RNA-seq analysis

We extracted RNA using a total RNA purification micro kit (Norgen cat.35300, Canada). We treated the lysate on the column with DNase I to remove DNA contamination. We extracted RNA from the three differentiation stages i.e., days 0, 26, and 39 in triplicates, and libraries were prepared by Illumine kits. The libraries were sequenced on Hi-Seq 2000 with 40 M reads per sample. We defined a TF degree of expression as follows: if the level of expression increases by at least 2-fold from Day 0 to Day 26, and the level of expression decreases/does not change from Day 26 to Day 39, then the TF is transcribed on day 26; otherwise, the TF was considered as not transcribed. This definition is used as a filter to eliminate TF binding to the REs of neuronal progenitors.

### Extant methods for *de-novo* motif discovery

We compared the motifs our framework detected to motifs found by well-known and established motif finders. The MEME-suite toolkit includes methods for *de novo* motif discovery. Given two sets of nucleotide sequences Multiple EM for Motif Elicitation (MEME) (Bailey and Elkan, 1994; Bailey et al., 2006) and Discriminative Regular Expression Motif Elicitation (DREME) (Bailey, 2011) find enriched motifs in one set compared to the other. We used MEME and DREME to find enriched motifs in the active enhancer compared to the non-active enhancer set and *vice versa*. BaMM webserver uses a probabilistic method for *de novo* motif discovery (Siebert and Soding, 2016; Kiesel et al., 2018). We used BaMM to discover enriched motifs in nucleotide sequences compared to a background model. An extra feature of BaMM is to compare the enriched motifs to motifs from known databases. The parameter settings and details on running the methods are described in Supplementary Information.

### Transgenic enhancer assays

We designed primers to amplify candidate sequences of REs from human genomic DNA (Supplementary Table S9). We cloned PCR products into the E1b-GFP-Tol2 enhancer assay vector containing an E1b minimal promoter followed by the green fluorescent protein (GFP) reporter gene. We injected these constructs into zebrafish embryos using standard procedures. For statistical significance, we injected at least 100 embryos per construct in at least two different injection experiments along with Tol2 mRNA to facilitate genomic integration. We observed and annotated GFP expression at 24, 48, and 72 hpf). We compared the annotation of the GFP expression driven by the tested minimal enhancer sequences to the GFP expressed pattern. We annotated the GFP expression using a Stereo Discovery V12 fluorescence stereomicroscope (Zeiss).

### Site-directed mutagenesis by overlap extension using the polymerase chain reaction

We generated site-directed deletions of predicted ZEB1 and CTCF binding sites on an e1b-GFP-Tol2 plasmid containing RE1 or RE6. Specific primers with the desired mutations (Supplementary Table S10) were designed to amplify the entire plasmid template using a PCR protocol. We removed the parent template using DpnI (methylation-dependent endonuclease) (NEB, #R0176) and transformed bacteria with the nuclease-resistant nicked plasmid (the PCR product). We isolated plasmids from the resulting colonies and screened them for the desired modification. We verified the positive clones by Sanger sequencing for the desired modification and the absence of additional modifications.

## Data Availability

Raw data are available via GEO database accession number GSE218668. Complete results are available in Supplementary Tables. The code used in this study is available at github.com/orensteinLab/DLFMoD.
