# Supplementary material for: Deciphering transcription factors and their corresponding regulatory elements during inhibitory interneuron differentiation using deep neural networks
Source: Front Cell Dev Biol. 2023 Feb 20;11:1034604. doi: 10.3389/fcell.2023.1034604 (PMC9986276; doi:10.3389/fcell.2023.1034604)
Supplement: Supplementary file 11 [file DataSheet1.docx]

**Supplementary Information**


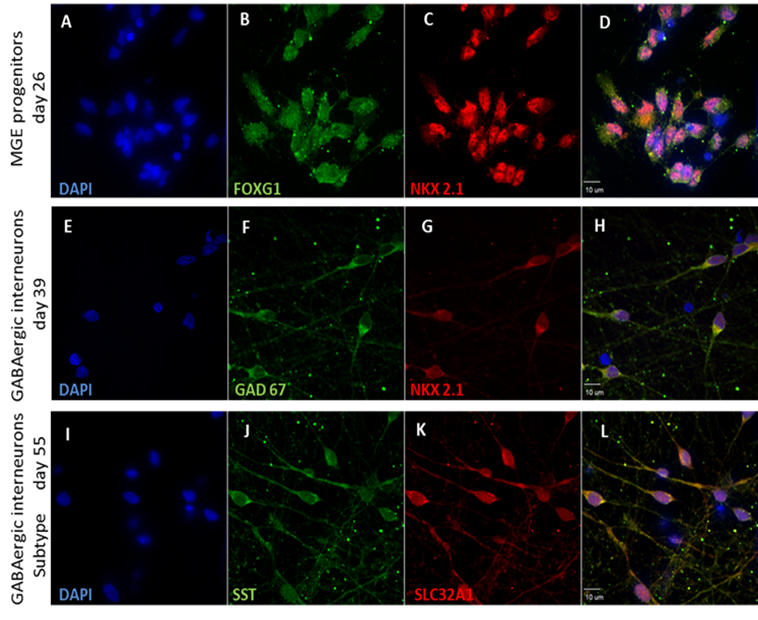


**Supplementary Figure S1. Immunofluorescence staining during differentiation of GABAergic-like interneurons.** (A-D) Immunostaining with FOXG1 and NKX2-1 that marked MGE progenitors at day 26 of differentiation; (E-H) Immunostaining with GAD67 and NKX2-1 that marked GABAergic interneurons at day 39 of differentiation. (I-L) Immunostaining with Somatostatin (SST) and SLC32A1 that both marked subtype of GABAergic-like interneurons at day 55 of differentiation. The nucleus is marked by DAPI staining (A, E, I).

**GC-content comparison**

As enriched sequence motifs may be influenced by different nucleotide content in the foreground and background sequences, we calculated the GC content of each set to check if there is a nucleotide frequency bias which may lead to spurious motifs. We calculated the frequency of G/C nucleotides in each sequence, in both active and non-active enhancer datasets. We applied Wilcoxon rank-sign test (Gehan, 1965) for comparing dataset frequencies to show that the difference in GC-frequency distribution is statistically significant (p-value<0.05). We found a significant difference in GC content between active and non-active (**Supplementary Figure S2**).


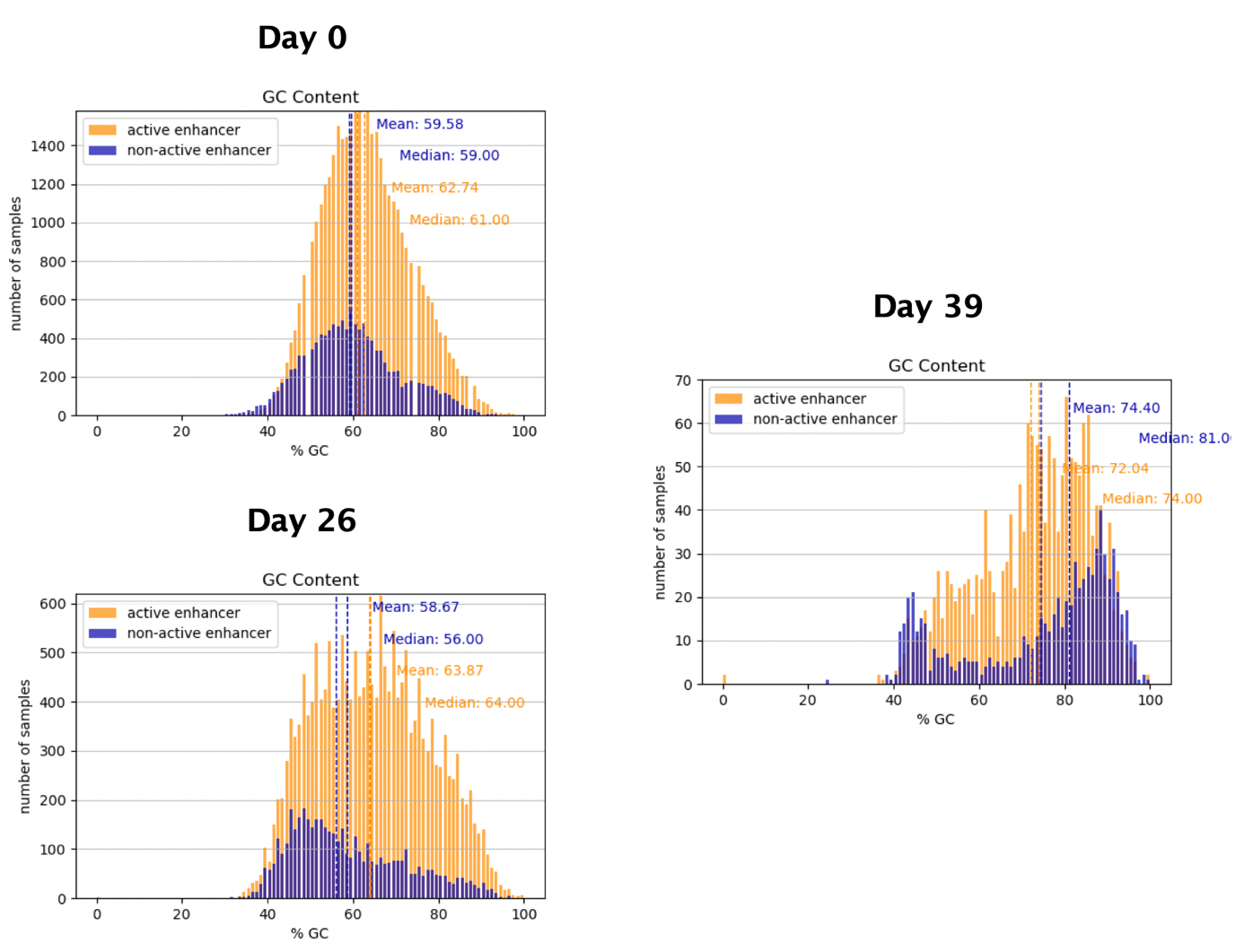


p-value = 5.62E-9

p-value = 7.45E-8

p-value = 2.64E-13

**Supplementary Figure 2. GC-content comparison.** Two histograms per differentiation stage (Day 0, Day 26, and Day 39) represent the distribution of GC content per sequence of active (positive) and non-active (negative) enhancers. P-values were calculated by a Wilcoxon rank-sign test.

**Model training and testing**

To evaluate the performance of the trained models, we used AUC (area under the receiver operating characteristic curve), a common metric for evaluating the binary classification (Bradley, 1997). AUC ranges in value from 0 to 1, with 0.5 achieved by a random guess. We chose the hyper-parameters of the network after training all combinations of the parameters (grid-search) listed below. The chosen hyper-parameters are those that yielded the maximum average AUC over 10-fold cross-validation (Rodríguez *et al.*, 2010). In each iteration, one part is used for testing and the nine other parts for training.

We constructed separate train and test sets for motif extraction and evaluating prediction performance. In evaluating prediction performance, we split the dataset into the train, and test sets. Chromosome 1 samples were the test set, and the other chromosomes were the train set. Following hyper-parameter selection, we trained the model with the chosen hyper-parameters and reported results on the test set. In the motif extraction task, we trained multiple models on the entire dataset. Then, we chose the model with achieving the highest AUC .

**Hyper-parameters search space**

| Hyper-parameter | Parameter space |
| --- | --- |
| Number of filters | 32, 64, 128, 256, 512, 1024, 2048 |
| Filter size | 4, 5, 6, 7, 8, 9 |
| Pooling size | 16, 32, Global Max Pooling |
| Batch size | 32, 64, 128, 256, 512, 1024 |
| Epochs | 15, 20, 30, 40 |

We applied grid-search to find optimal hyper-parameters with respect to the average AUC achieved in 10-fold cross-validation. The optimal parameters are marked in red.

**Trained deep neural network performance**

We trained a CNN for binary classification of active compared to non-active enhancers. We performed extensive hyper-parameters search by evaluating model performance in 10-fold cross-validation. The hyper-parameters marked in red achieved the highest AUC of 0.72±0.01 (Figure S3A). Then, we trained a model with these hyper-parameters. The model achieved an AUC of 0.71±0.00 and succeeded in classifying a sequence into one of the active or non-active enhancer classes (Figure S3B). To compare to a base-line approach, we trained a linear regression model on the same input. The linear regression model achieved an AUC of only 0.53, emphasizing the need for complex models, such as CNNs.

As evolutionary conversion may imply the function of DNA sequences as REs (Hardison *et al., 2000*; Pennacchio, L.A. *et al.* *2006*), we augmented the sequence input by phastCons score and trained a CNN model after searching for optimal hyper-parameters (Siepel *et al., 2005*). We simply appended the phastCons score vector to the one-hot-encoded sequence matrix and evaluated model performance by the average AUC in 10-fold cross-validation. We found that the model improved in AUC only by +0.01, +0.02, +0.01 for the three differentiation stages (Day0, Day 26, and Day 39, respectively), suggesting, that both active and non-active REs can be conserved through evolution. Thus, due to the minor improvement using phastCons and the infeasibility to incorporate it into the extant version of TF-MoDISco, we decided not to include evolutionary conservation as additional information to our framework.

*
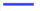

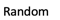
*


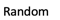

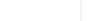

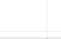

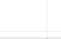

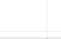


B

A


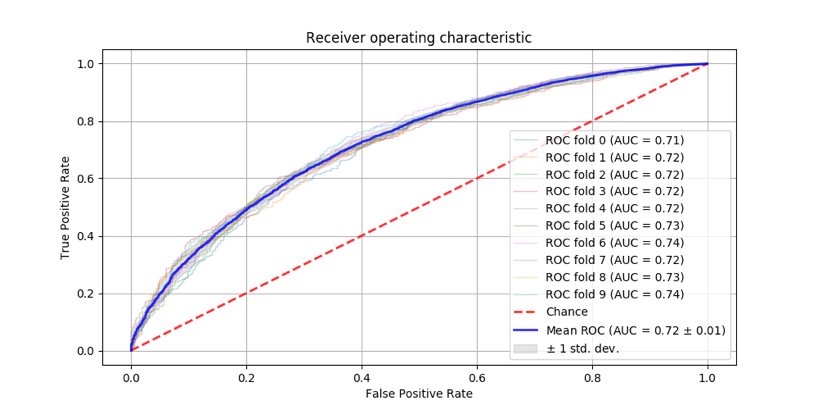

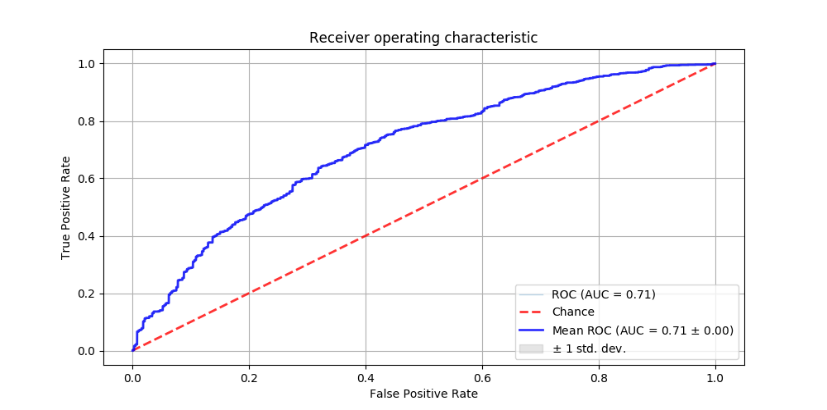


A

B

**
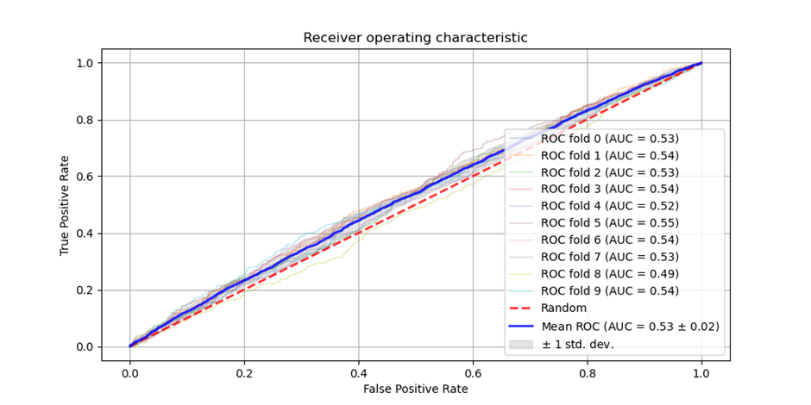
**

C

**Supplementary Figure S3. The ROC curves of the trained model in 10-fold cross-validation and on a held-out test set.** (A) We evaluated model performance using 10-fold cross-validation for hyper-parameters search leaving out chromosome 1. (B) Performance evaluation on a held-out test set. Training with the chosen hyper-parameters on all samples from all chromosomes but chromosome 1, which was used as the test set. (C) Performance evaluation of a of linear regression approach.

**MEME suite for motif post analysis**

The most popular toolkit for motif discovery and analysis is the MEME-suite (Bailey et al., 2015). We give brief details of the different methods of the MEME-suite used in our motif post-analysis. We used AME (McLeay and Bailey, 2010) to test the enrichment of a given motif in the active enhancer compared to the non-active enhancer set. We applied AME with the default parameters settings. The TOMTOM tool (Gupta *et al.*, 2007; Benjamini and Hochberg, 1995) calculates the similarity of a given motif to a database of target motifs. The MEME-suite includes publicly available TF motif databases. TOMTOM calculates statistical information to help in finding potential TFs that can bind to a given set of motifs. We applied TOMTOM with parameter settings, “-min-overlap 5 -mi 1 -dist ed -evalute -thresh 10.0”. While running TOMTOM, a library of known motifs was used from three MEME-suite motif datasets: (i) JASPAR2018 CORE vertebrates NON-REDUNDANT (*in vivo* and *in silico*) (Khan *et al.*, 2018), (ii) UniPROBE Mouse (protein-binding microarray) (Newburger and Bulyk, 2009), (iii) Jolma2013 Human and Mouse (HT-SELEX) (Jolma *et al.*, 2013). These three MEME-suite motif databases cover a comprehensive list of human and mouse TFs.

**Running extant methods for *de-novo* motif discovery**

1. Multiple EM for Motif Elicitation tool (MEME) (Bailey et al., 2006; Bailey and Elkan, 1994) - Given two sets of nucleotide sequences, MEME finds enriched motifs in one set compared to the other. We used MEME to find enriched motifs in the positive set sequences compared to negative set sequences (“-objfun de”). We did not assume that a sequence contains at most one occurrence of each motif (“-mod anr”), and we enabled MEME to find motifs shorter than 8nt (“-minw 6”).
2. Discriminative Regular Expression Motif Elicitation (DREME) (Bailey, 2011) - Given two sets of nucleotide sequences, DREME finds enriched motifs in one set compared to the other. We used DREME to find enriched motifs in the active enhancer set compared to the non-active enhancer set with default parameter settings.
3. BaMM webserver uses a probabilistic method for *de-novo* motif discovery (Kiesel *et al.*, 2018; Siebert and Söding, 2016). BaMM webserver discovers enriched motifs in nucleotide sequences compared to a background model. Then, it compares the enriched motifs to motifs from known databases. We applied BaMM with parameter settings: (i) MMcompare Motif Database: JASPAR2018, (ii) Background Sequences: <the negative set>, (iii) Background Model Order: 0, (iv) IUPAC Optimization Score: ENRICHMENT.

MEME has higher specificity (a higher proportion of negative samples that are correctly classified) than DREME, but this comes at the cost of longer run times (Ma *et al.*, 2014). MEME can only find a single enriched motif at a time, while DREME can find multiple enriched motifs in parallel (Tran and Huang, 2014). DREME is limited to a maximum motif length of 8.

**Comparison to enriched motifs found by extant methods**

To detect TF motifs in the putative enhancers, we ran three *de-novo* motif-finding tools: MEME (Bailey et al., 2006; Bailey and Elkan, 1994), DREME (Bailey, 2011), and BaMM (Kiesel et al., 2018; Siebert and Söding, 2016). We used the tools to detect TF motifs in the putative enhancers, where the non-active enhancer set was used as the primary and active enhancer set as the control and vice versa. Subsequently, we examined the output to test if these tools found putative regulatory motifs. In addition to motif discovery, BaMM compares the discovered motifs to a database of known motifs. However, MEME and DREME tools do not have this feature, and therefore, we applied TOMTOM on each discovered motif in these tools to find the similarity of known motifs.

Interestingly, the outputs of these three tools result in different and varied enriched motifs **(Supplementary Figure S4)**. MEME tool found four enriched motifs, where three enriched motifs were found in non-active compared to active enhancers, and one enriched motif was in active compared to non-active enhancers. BaMM tool found five enriched motifs, where three enriched motifs were found in non-active compared to active enhancers and two enriched motifs in active compared to non-active enhancers. DREME tool found 61 enriched motifs, where 39 enriched motifs were found in non-active compared to active and 22 enriched motifs in active compared to non-active **(Supplementary Table S3)**.

A common disadvantage to all methods is the parameter space. Additionally, DREME has a limitation on the produced TF motif length to a maximum of 8 nt. Our method found three TFs (NHLH1/ASCL1, SOX4/11, NEUROG2, ZEB1, CTCF, and NR2F1) related to the process of cell differentiation in brain cells, while DREME produced one TF (ZEB1), and MEME and BaMM yielded poor results of zero relevant motifs. To conclude, our framework discovered more biologically relevant motifs compared to the extant methods.

**
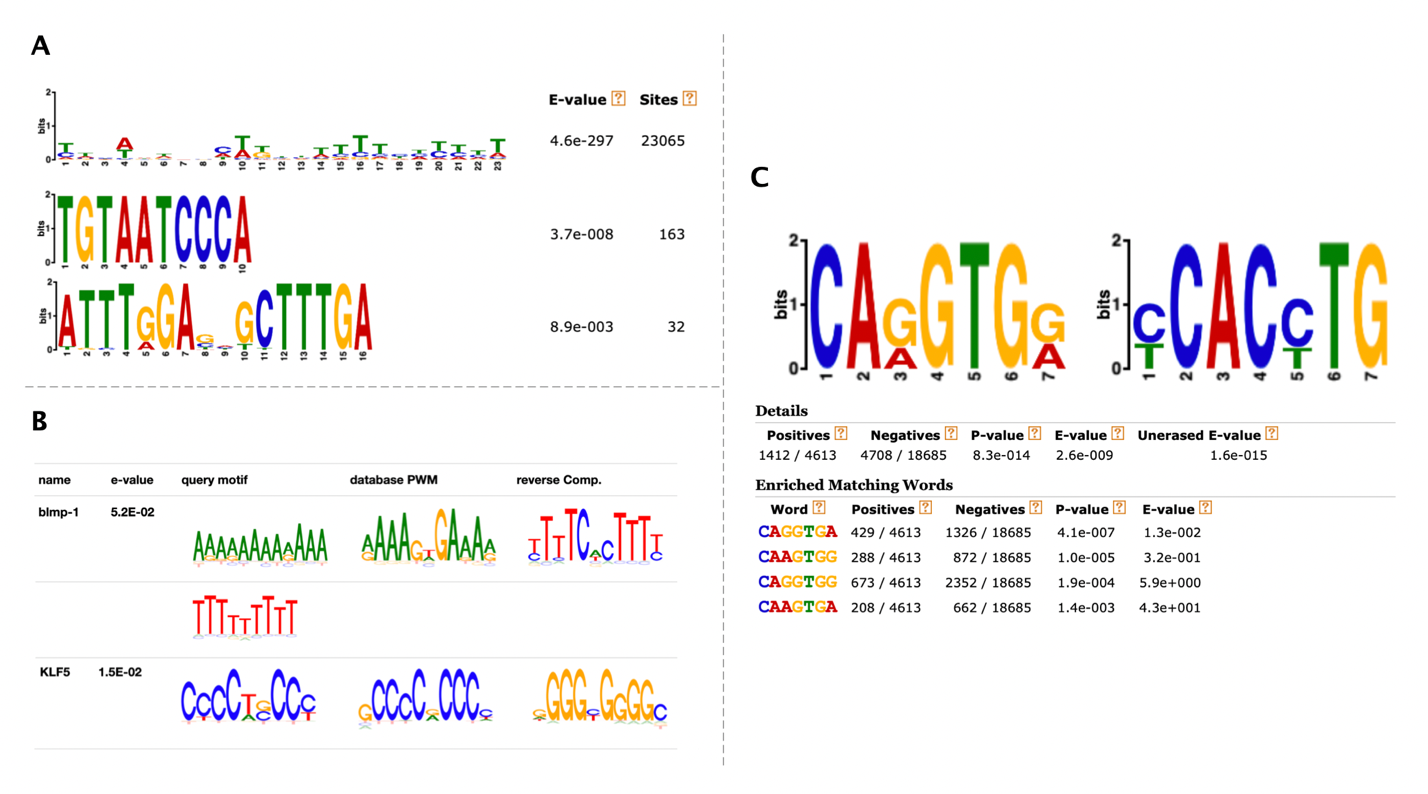
**

**Supplementary Figure S4.** The results of three extant motif-finding methods, MEME, BaMM, and DREME, when applied to the negative set vs. the positive set. (A) The set of enriched motifs discovered by MEME. The motifs were disqualified because they did not pass the overlap > 70% criteria. (B) The set of enriched motifs discovered by BaMM: the motifs were disqualified because they did not pass bias < 80% criteria. (C) ZEB1 motif is the only non-spurious (i.e., fulfilled the guideline criteria) motif out of 39 motifs discovered by DREME.

**
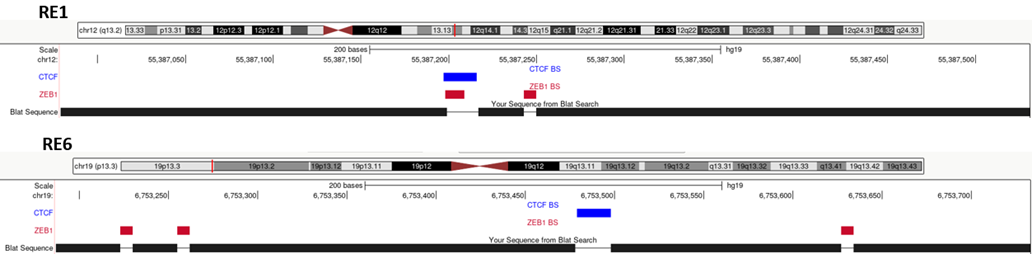
**

**Supplementary Figure S5. Deletions of ZEB1 and CTCF predicted binding sites in RE1 and RE6 sequences.** Red bars indicate ZEB1-predicted binding sites, and blue bars indicate CTCF-predicted binding sites. DNA sequences of RE1 and RE6 are indicated in black boxes. The gaps indicate the deletions generated using site-directed mutagenesis.

**References**

*Bailey,T.L. (2011) DREME: Motif discovery in transcription factor ChIP-seq data. Bioinformatics.*

*Bailey,T.L. and Elkan,C. (1994) Fitting a mixture model by expectation maximization to discover motifs in biopolymers. Proc. Int. Conf. Intell. Syst. Mol. Biol.*

*Bailey,T.L. et al. (2006) MEME: Discovering and analyzing DNA and protein sequence motifs. Nucleic Acids Res.*

*Bailey,T.L. et al. (2015) The MEME Suite. Nucleic Acids Res.*

*Benjamini,Y. and Hochberg,Y. (1995) Controlling the False Discovery Rate: A Practical and Powerful Approach to Multiple Testing. J. R. Stat. Soc. Ser. B.*

*Bradley,A.P. (1997) The use of the area under the ROC curve in the evaluation of machine learning algorithms. Pattern Recognit.*

*Gehan,E.A. (1965) A generalized Wilcoxon test for comparing arbitrarily singly-censored samples. Biometrika.*

*Gupta,S. et al. (2007) Quantifying similarity between motifs. Genome Biol.*

*Hardison, R.C. Conserved noncoding sequences are reliable guides to regulatory elements. Trends Genet 16, 369-72 (2000).*

*Heinz,S. et al. (2010) Simple Combinations of Lineage-Determining Transcription Factors Prime cis-Regulatory Elements Required for Macrophage and B Cell Identities. Mol. Cell.*

*Jolma,A. et al. (2013) DNA-binding specificities of human transcription factors. Cell.*

*Khan,A. et al. (2018) JASPAR 2018: Update of the open-access database of transcription factor binding profiles and its web framework. Nucleic Acids Res.*

*Kiesel,A. et al. (2018) The BaMM web server for de-novo motif discovery and regulatory sequence analysis. Nucleic Acids Res.*

*Ma,W. et al. (2014) Motif-based analysis of large nucleotide data sets using MEME-ChIP. Nat. Protoc.*

*McLeay,R.C. and Bailey,T.L. (2010) Motif Enrichment Analysis: A unified framework and an evaluation on ChIP data. BMC Bioinformatics.*

*Newburger,D.E. and Bulyk,M.L. (2009) UniPROBE: An online database of protein binding microarray data on protein-DNA interactions. Nucleic Acids Res.*

*Pennacchio, L.A. et al. In vivo enhancer analysis of human conserved non-coding sequences. Nature 444, 499-502 (2006).*

*Rodríguez,J.D. et al. (2010) Sensitivity Analysis of k-Fold Cross Validation in Prediction Error Estimation. IEEE Trans. Pattern Anal. Mach. Intell.*

*Siebert,M. and Söding,J. (2016) Bayesian Markov models consistently outperform PWMs at predicting motifs in nucleotide sequences. Nucleic Acids Res.*

*Siepel, A. et al. Evolutionarily conserved elements in vertebrate, insect, worm, and yeast genomes. Genome Res 15, 1034-50 (2005).*

*Tran,N.T.L. and Huang,C.H. (2014) A survey of motif finding Web tools for detecting binding site motifs in ChIP-Seq data. Biol. Direct.*

*Wang,H. et al. (2019) ZEB1 Represses Neural Differentiation and Cooperates with CTBP2 to Dynamically Regulate Cell Migration during Neocortex Development. Cell Rep.*

*Wang,Z. et al. (2009) RNA-Seq: A revolutionary tool for transcriptomics. Nat. Rev. Genet.*
